# Hepatic Artery Pseudoaneurysm Following Gunshot Injury With Early Rupture

**DOI:** 10.7759/cureus.15866

**Published:** 2021-06-23

**Authors:** Mohamed Ahmed, Mohamed Elkahly, Stephen Dada, Ahmed Mahmoud, Michael Chin

**Affiliations:** 1 Surgery, University of California, Riverside, Riverside, USA; 2 Surgery, Riverside Community Hospital, Riverside, USA; 3 General Surgery, Universal Health Services, Temecula, USA; 4 Surgery, Riverside Community Hospital/University of California, Riverside, Riverside, USA

**Keywords:** gsw, pseudoaneurysm, penetrating liver trauma, aneurysm rupture, penetrating injuries, thoracoabdominal trauma

## Abstract

Pseudoaneurysms are a rare and potentially life-threatening complication that can be caused by trauma, infections, tumors, autoimmune diseases, organ transplants, or idiopathic causes. The management of liver trauma is based on the anatomy of the injury and the patient’s physiology. Posttraumatic hepatic artery pseudoaneurysm (HAP) is a life-threatening complication that requires prompt recognition and a multidisciplinary approach in its management. We present a case of HAP rupture two weeks following a gunshot wound to the liver.

## Introduction

Arterial pseudoaneurysms result from damage to the arterial wall consisting of a contained hematoma with turbulent blood flow and a neck that very rarely closes spontaneously [1]. Femoral artery pseudoaneurysms are the most common in clinical practice, with an incidence of 0.6-4.8% following femoral artery access for endovascular procedures [2]. The incidence of hepatic artery pseudoaneurysm (HAP) after penetrating trauma is estimated at approximately 0.001% with a high risk of spontaneous rupture and reported mortality rates of 25% and 70% [3,4]. HAP has been reported as a complication following laparoscopic cholecystectomy and orthotopic liver transplantation [5,6].

## Case presentation

**Figure 1:**
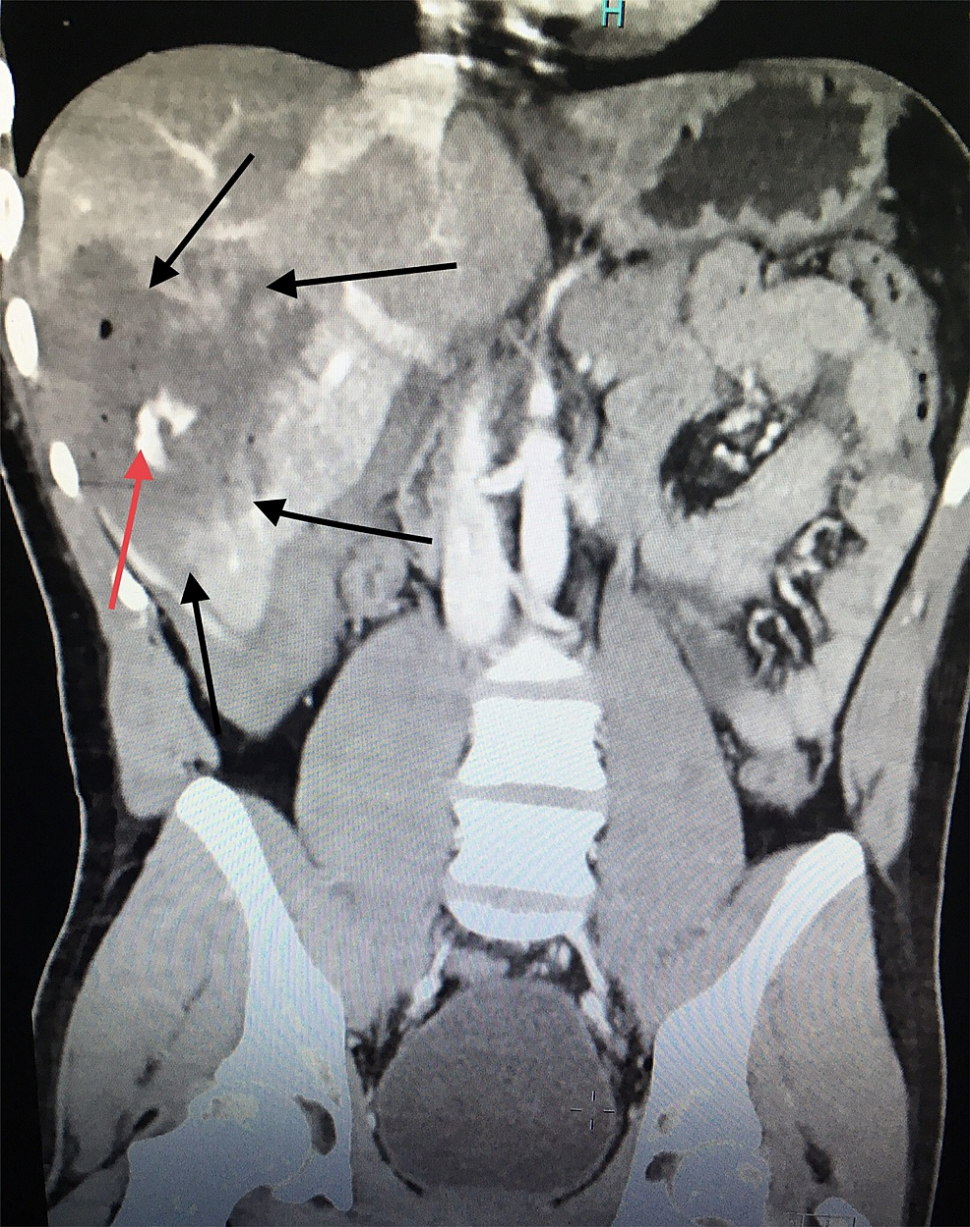
CT angiogram of the abdomen (coronal plane). Pseudoaneurysm: contrast outside the vessel depicted by the red arrow. Grade IV liver injury depicted by the black arrow.

A 23-year-old male presented to our emergency room with acute abdominal pain and lightheadedness. The patient had sustained a right thoracoabdominal gunshot wound two weeks earlier and was managed nonoperatively with a right chest tube thoracostomy. The evaluation revealed a low-grade fever of 99°F, tachycardia with a pulse rate of 100 beats per minute, blood pressure of 105/60 mmHg, respiratory rate of 20 breaths per minute, and oxygen saturation of 99% on room air. Abnormal laboratory findings included hemoglobin of 5.8 g/dL (normal: 13.8-17.2 g/dL), white cell count of 1,492/µL (normal: 4,500-11,000/µL), total bilirubin of 1.5 mg/dL (normal: 1.2 mg/dL), alanine aminotransferase of 105 U/L (normal: 29-33 U/L), and aspartate aminotransferase of 75 U/L (normal: 5-40 U/L). Computerized tomography (CT) angiogram demonstrated a grade IV liver injury, hemoperitoneum, and a pseudoaneurysm of the right hepatic artery with active extravasation (Figure 1).

**Figure 2:**
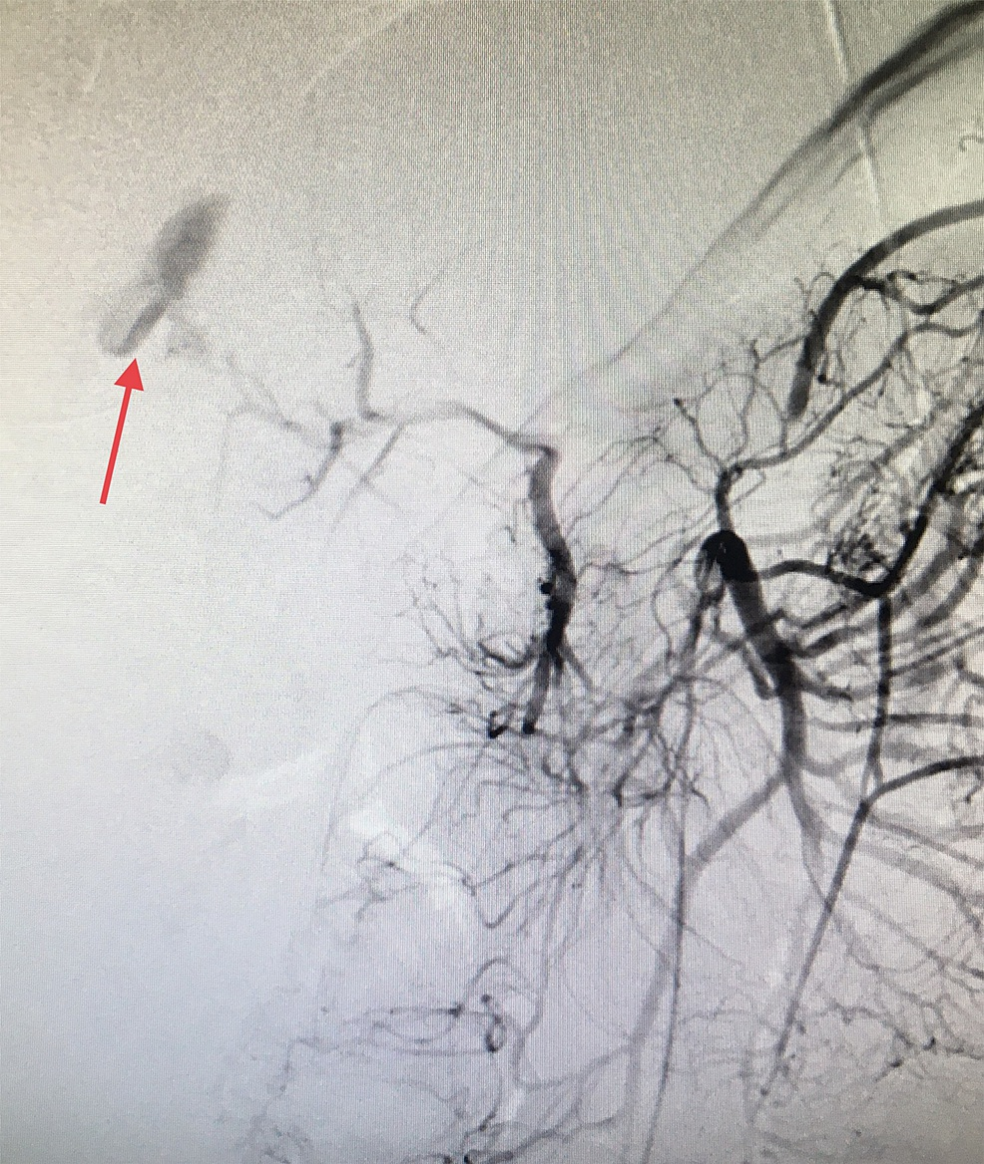
Angiography. Contrast extravasation consistent with the right hepatic artery pseudoaneurysm (red arrow).

**Figure 3:**
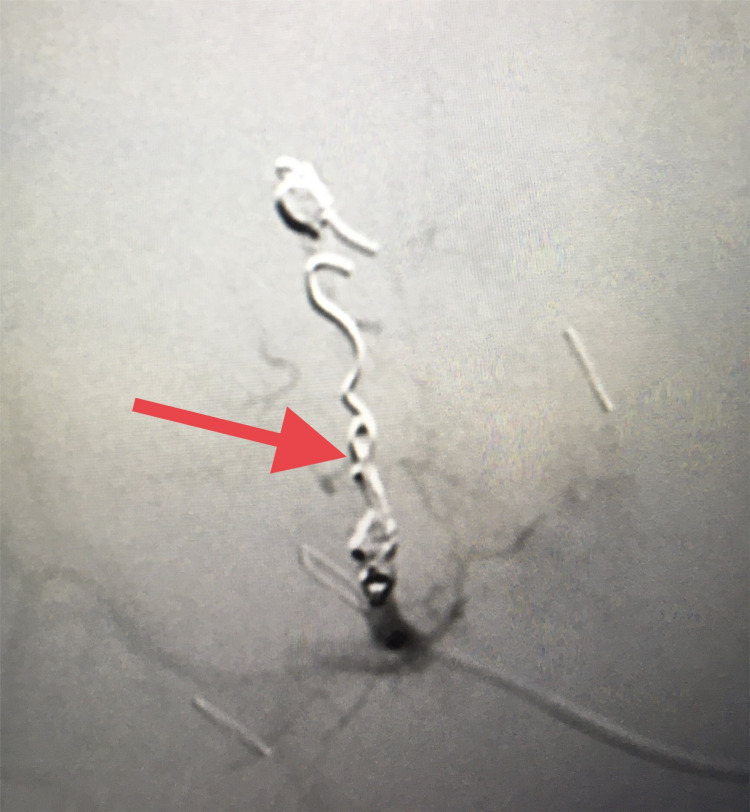
Embolization of right hepatic artery pseudoaneurysm (red arrow depicts the coils).

The patient was taken to the operating room (in the absence of an in-house interventional radiologist and a clinical picture concerning hemorrhagic shock) and an exploratory laparotomy was performed. Gross hemoperitoneum was evacuated, active bleeding from a friable right lobe of the liver due to his recent injury was controlled with packs applied above and below the liver, and a temporary vacuum closure device was applied. The patient was transferred to the interventional radiology suite and underwent successful coil embolization of the right HAP (Figures 2, 3).

The patient was taken back to the operating room on postoperative day two for a planned re-exploration. The packs were removed, drains were inserted for a noticeable bile leak, and wound closure was done. The patient did well and was eventually discharged from the hospital in a stable condition. Follow-up in our clinic was uneventful.

## Discussion

Pseudoaneurysms result from damage to the arterial wall as a result of trauma or iatrogenic complications following surgery or interventional access with an incidence of up to 4.8% with femoral artery access [7]. The exact incidence of traumatic pseudoaneurysms is difficult to ascertain and can result from blunt or penetrating trauma [8]. Infection as a cause of pseudoaneurysms is characterized by rapid expansion leading to rupture [9]. Vasculitis, such as in the case of Behçet’s syndrome, polyarteritis nodosa, systemic lupus erythematosus, giant cell arteritis, or Takayasu’s arteritis, can be complicated with pseudoaneurysm formation [10]. Vessel erosion from benign or malignant tumors can result in a pseudoaneurysm [11].

HAP is rare and has been reported as a complication of acute pancreatitis, interventional radiology procedures, and surgical procedures including laparoscopic cholecystectomy [12]. Spontaneous HAP rupture is very rare and has been reported in a patient with systemic lupus erythematosus and autoimmune hepatitis [13,14]. HAP rupture following blunt trauma has also been reported in children [15]. A seven-year retrospective study at the University of California, Irvine Medical center found five cases of hepatic artery aneurysms among 18,015 trauma and surgical admissions (blunt abdominal trauma, liver biopsy, pancreatic pseudocyst, and polyarteritis nodosa), representing an incidence of 0.03%, in addition to two cases among 200 orthotopic liver transplants [16]. The incidence increases with higher-grade liver injury (III-IV), with a median identification time of 6.5 days after the injury [17]. The clinical presentation of HAPs may vary from incidental detection to an emergency presentation with rupture. Hemoperitoneum results from extrahepatic rupture, while gastrointestinal bleeding caused by hemobilia is a consequence of rupture into the biliary tree. Once HAP is diagnosed, a definite intervention, regardless of the size, is warranted as the clinical course of these aneurysms is unpredictable with a possibility of rupture and acute hemodynamic compromise [18]. Diagnosis can be made with different radiological modalities, with ultrasound, contrast-enhanced CT scan, CT angiography, contrast-enhanced MRI, and selective angiography being the most valuable investigation modalities for the diagnosis of HAP with a sensitivity of 100% [19]. Incidentally detected HAP can be treated with angiographic stent placement or embolization if feasible. Surgical ligation and resection with or without reconstruction can also be employed. HAP rupture is better managed with angioembolization if patient hemodynamics permits and interventional radiology service is promptly available. In a hemodynamically unstable patient, surgical exploration with affected vessel ligation can be life-saving [20]. In our case, the liver injury following the gunshot wound made it difficult to localize the bleeding vessel as the liver tissue was friable. Hence, bleeding control and hemodynamic stability were achieved with appropriate packing, followed by angioembolization of the injured vessel. In our case, selective embolization was not feasible and right hepatic artery coil embolization was performed.

## Conclusions

HAP is a rare but potentially fatal complication of liver injury. Prompt diagnosis and treatment are necessary to avoid the potential morbidity and mortality associated with rupture and hemorrhage. Transcatheter embolization plays a significant role in the treatment of symptomatic and ruptured HAP. The incidence of HAP increases with higher-grade liver injury. Aggressive surveillance for HAP with interval CT angiography five to seven days post-injury may be warranted, especially for grade IV and V injuries.
